# Prevalence and risk factors of MRI-defined brain infarcts among Chinese adults

**DOI:** 10.3389/fneur.2022.967077

**Published:** 2022-10-12

**Authors:** Jing Wu, Yongxiang Gao, Vasanti Malik, Xiang Gao, Ruiqi Shan, Jun Lv, Yi Ning, Bo Wang, Liming Li

**Affiliations:** ^1^Department of Epidemiology and Biostatistics, School of Public Health, Peking University Health Science Center, Beijing, China; ^2^Meinian Institute of Health, Beijing, China; ^3^Department of Nutritional Sciences, University of Toronto, Toronto, ON, Canada; ^4^Department of Nutritional Sciences, The Pennsylvania State University, University Park, State College, PA, United States; ^5^Meinian Public Health Institute, Peking University Health Science Center, Beijing, China; ^6^School of Public Health, Hainan Medical University, Haikou, China

**Keywords:** prevalence, risk factors, MRI, brain infarcts, epidemiological study

## Abstract

**Background:**

Few studies have explored the prevalence and risk factors of brain infarcts (BI) detected by magnetic resonance imaging (MRI) in China. The purpose was to evaluate the prevalence and risk factors of brain infarcts (BI) detected by magnetic resonance imaging (MRI) in 1.4 million Chinese adults.

**Methods:**

This was a multicenter cross-sectional study conducted on 1,431,527 participants aged ≥18 years (mean age: 46.4 years) who underwent MRI scans in health examinations from 28 provinces of China in 2018. MRI-defined BI was defined as focal parenchymal lesions ≥3 mm. Multivariable logistic regression analyses were performed to evaluate risk factors associated with MRI-defined BI.

**Results:**

The age- and sex-standardized prevalence of MRI-defined BI, lacunar and non-lacunar infarcts were 5.79% (5.75–5.83%), 4.56% (4.52–4.60%), and 1.23% (1.21–1.25%), respectively. The sex-standardized prevalence of MRI-defined BI ranged from 0.46% among those aged 18–29 years to 37.33% among those aged ≥80 years. Men (6.30%) had a higher age-standardized prevalence of MRI-defined BI than women (5.28%). The highest age- and sex-standardized prevalence of MRI-defined BI was observed in the Northwest (8.34%) and Northeast (8.02%) regions, while the lowest prevalence was observed in the Southwest (4.02%). A higher risk of MRI-defined BI was associated with being male [odd ratio (OR) 1.17, 95% CI 1.15–1.19], older age (OR per 10-year increments 2.33, 2.31–2.35), overweight (1.12, 1.10–1.14) or obesity (1.18, 1.16–1.21), hypertension (1.80, 1.77–1.83), diabetes (1.24, 1.21–1.26), and dyslipidemia (1.07, 1.05–1.08).

**Conclusion:**

MRI-defined BI is highly prevalent in China, even among young adults. MRI-defined BI was associated with being male, older age, living in the northern region, and metabolic conditions.

## Introduction

China currently carries the world's largest burden of stroke, which has become a major public health challenge (1, 2). However, overt stroke, easily recognized clinically, represents only the tip of the iceberg. In contrast, silent brain infarcts (SBI) are often ignored and represent the larger below the surface of the water (3). With the development of brain-imaging techniques, brain abnormalities are commonly found using brain magnetic resonance imaging (MRI) (4, 5). The prevalence of MRI-defined BI exceeds, by far, the prevalence of symptomatic stroke (6). Although the majority of MRI-defined BI were covert without clinical stroke symptoms (7), they are highly valuable in predicting subsequent risk of symptomatic stroke, dementia, and mortality (8, 9). However, few studies regarding the epidemiology of MRI-defined BI have been conducted in China, and previous studies were limited by small sample sizes or certain geographic regions only (7, 10). Meanwhile, the association among age, hypertension, and MRI-defined BI has been widely accepted, but the association between obesity, dyslipidemia, and MRI-defined BI remains unclear (10).

The total number of people undergoing routine health examinations in China reached 575 million in 2018, accounting for 42% of the total population of China (11). It would be of great interest to explore the epidemiology of MRI-defined BI for early prevention and control of stroke and dementia. Therefore, we conducted this study to investigate the prevalence and risk factors of MRI-defined BI among 1.4 million participants who underwent MRI scans.

## Methods

### Study design and participants

The study was a nationwide, multicentric, population-based study using data from the Meinian Onehealth Healthcare, which is a largest health screening organization covering nearly all provinces on Chinese mainland. Descriptions of the database have been reported previously (12, 13). Each health screening center provides annual routine health examinations to its members. A unified standard examination protocol was established in each center. In fact, most of the health examinations in the health screening centers were paid for by companies or group and provided to employees free of charge as a kind of welfare. Whether to do a brain MRI scan or not was determined by the company or group, not based on their pre-clinical or clinical symptoms or other risk factors. We extracted participants who had MRI scans from the whole database. From January 1, 2018 to December 31, 2018, a total of 1,442,518 participants without contraindications to MRI underwent a brain MRI. For those who attended more than two health examinations, only the most recent checkup data were included. We excluded participants < 18 years of age, and those with missing data on age, sex, and health screening center, leaving 1,440,738 participants for analysis. For the stability of the results, we further excluded data from newly opened 71 health screening centers (< 500 participants for a brain MRI). A total of 1,431,527 participants (725,261 men and 706,266 women) aged ≥18 years from 254 health screening centers in 161 cities in 28 provinces of China were included in the final analysis.

The study was approved by the Peking University Institutional Review Board with a waiver of informed consent (IRB00001052-19077). Identifiable data of participants were removed and only unidentifiable data was used for the study.

### Assessment of MRI-defined brain infarcts

Each participant underwent MRI scans in adherence to a standardized scan protocol. The MRI scans were performed by a certified imaging technician at each health screening center. All brain scans were performed on a 1- or 1.5-T MRI scanner (more than 85% of MRI scanners in health screening centers are 1.5-T scanners), which comprised at least T1 weighted images, T2 weighted images, and proton density or fluid attenuated inversion recovery (FLAIR) sequences. MRI images were read by one experienced radiologist and confirmed by another experienced radiologist in each health screening center, and any disagreements were solved by consensus through discussion. These radiologists were blinded to clinical and demographic data. Infarcts on MRI scans were defined as an area of abnormal signal intensity in a vascular distribution that lacked mass effect with a size ≥3 mm (7, 14). Lacunar infarcts were distinguished from Virchow-Robin spaces based on their irregular shape, non-vascular appearance, and presence of a hyperintense rim. According to the criteria of the standards for reporting vascular changes on neuroimaging (STRIVE) (4), lacunar infarcts were defined as focal lesions of < 15–20 mm in diameter in the territory of penetrating arteries, located in subcortical areas with the same signal characteristics as cerebrospinal fluid on all MRI-sequences, and other infarcts were considered as non-lacunar infarcts. All participants were categorized as having or not having at least 1 infarct. MRI-defined BI included lacunar infarcts and non-lacunar infarcts.

### Assessment of covariates

Face-to-face interviews were conducted by trained health professionals to collect information on the demographic characteristics and medical history of each participant. Body weight, height, and blood pressure were measured for all participants using standard methods. Overnight fasting blood samples for each participant were used to measure glucose and lipid levels.

We divided the mainland of China into seven geographic regions: northeast China, north China, northwest China, central China, east China, south China, and southwest China, which were divided based on the geographical divisions of China. Geographical variations in 12 leading risk factors related to cardiovascular disease (15) and in stroke burden in China have been reported previously (1).

Body mass index (BMI) was calculated as body weight (kg) divided by the square of height (m). Overweight (BMI ≥24.0 and ≤ 27.9 kg/m^2^) and obesity (BMI ≥28.0 kg/m^2^) were defined according to the BMI classification for Chinese adults (16). Hypertension was defined as systolic blood pressure ≥140 mm Hg, or diastolic pressure ≥90 mm Hg, having a history of hypertension or use of blood pressure lowering medications. Diabetes was determined by a fasting level of plasma glucose ≥7.0 mmol/L, having a history of diabetes, or use of antidiabetic medications. According to 2016 Chinese guidelines for the management of dyslipidemia in adults, dyslipidemia was defined as having any of the following: triglyceride level ≥2.3 mmol/L, total cholesterol level ≥6.2 mmol/L, high-density lipoprotein cholesterol level < 1.0 mmol/L, low-density lipoprotein cholesterol level ≥4.1 mmol/L, having a history of dyslipidemia, or use of lipid-regulating medications.

### Statistical analysis

The characteristics of study participants were presented as mean [standard deviation (SD)] for continuous variables, and percentages for categorical variables. The statistical significance of differences was performed using analysis of variance (ANOVA) for continuous variables and the Chi-square test for categorical variables. The prevalence and 95% confidence intervals (CI) standardized by age and sex were calculated among different sub-groups of characteristics, using the 2010 National Population Census as the standard population. The age-standardized prevalence was calculated by sex and the sex-standardized prevalence was also calculated by age group. Choropleth maps were produced using R software (version 3.6) to visually examine geographical variations in the prevalence of MRI-defined BI. The data illustrated in the maps were age-and sex-standardized prevalence with 95% CIs. Multivariable logistic regression analyses were conducted to investigate risk factors for MRI-defined BI adjusted for age, sex, geographical region, BMI, hypertension, diabetes, and dyslipidemia in the models.

All statistical analyses were performed using R version 3.6 (http://www.r-project.org/) and SAS version 9.4 (SAS Institute, Cary, NC). Statistical significance was defined as two-sided *P*-values < 0.05.

### Data availability

The data supporting the findings of this study are available from the corresponding author upon reasonable request.

## Results

### Characteristics of study participants

A total of 1,431,527 participants were included in the study. The characteristics of the study participants are shown in Table 1. The mean age of study participants was 46.4 (SD 12.4) years; approximately three-quarters (75.2%) were aged between 30 and 59 years and 50.7% (*n* = 725,261) were men. Nearly half of the participants (49.2%) had overweight or obesity, and 26.3, 7.0, 36.8% of the study participants had hypertension, diabetes, and dyslipidemia, respectively. Participants with MRI-defined BI were more likely to be older, male and had a higher prevalence of overweight or obesity, hypertension, diabetes, and dyslipidemia compared to those without MRI-defined BI (all *P* < 0.05).

**Table 1:** Characteristics of study participants by MRI-defined brain infarct status^a^.

**Characteristics**	**Overall**	**Without brain infarcts**	**Participants with MRI-defined brain infarcts**		
			**All MRI-defined brain infarcts**	**Lacunar infarcts**	**Non-lacunar infarcts**
*N*	1,431,527 (100)	1,331,282 (93.0)	100,245 (7.0)	79,724 (5.6)	20,521 (1.4)
**Sex**
Men	725,261 (50.7)	669,065 (50.3)	56,196 (56.1)	44,260 (55.5)	11,936 (58.2)
Women	706,266 (49.3)	662,217 (49.7)	44,049 (43.9)	35,464 (44.5)	8585 (41.8)
Age (years), mean ± SD	46.4 ± 12.4	45.5 ± 12.1	58.7 ± 10.4	58.2 ± 10.4	60.5 ± 10.0
**Age group**
18–29	135,597 (9.5)	134,969 (10.1)	628 (0.6)	567 (0.7)	61 (0.3)
30–39	315,480 (22.0)	312,379 (23.5)	3,101 (3.1)	2,699 (3.4)	402 (2.0)
40–49	381,901 (26.7)	368,880 (27.7)	13,021 (13.0)	10,992 (13.8)	2,029 (9.9)
50–59	380,033 (26.5)	344,435 (25.9)	35,598 (35.5)	28,890 (36.2)	6,708 (32.7)
60–69	174,489 (12.2)	140,480 (10.6)	34,009 (33.9)	26,194 (32.9)	7,815 (38.1)
70–79	37,092 (2.6)	25,823 (1.9)	11,269 (11.2)	8,430 (10.6)	2,839 (13.8)
≥80	6,935 (0.5)	4,316 (0.3)	2,619 (2.6)	1,952 (2.4)	667 (3.3)
**Geographical region**
Northeast	134,747 (9.4)	118,844 (8.9)	15,903 (15.9)	14,390 (18.0)	1,513 (7.4)
North China	117,393 (8.2)	107,176 (8.1)	10,217 (10.2)	6,106 (7.7)	4,111 (20.0)
Northwest	138,009 (9.6)	124,440 (9.3)	13,569 (13.5)	11,968 (15.0)	1,601 (7.8)
East China	448,345 (31.3)	423,178 (31.8)	25,167 (25.1)	19,779 (24.8)	5,388 (26.3)
Central China	263,787 (18.4)	243,430 (18.3)	20,357 (20.3)	14,897 (18.7)	5,460 (26.6)
South China	140,240 (9.8)	133,618 (10.0)	6,622 (6.6)	5,736 (7.2)	886 (4.3)
Southwest	189,006 (13.2)	180,596 (13.6)	8,410 (8.4)	6,848 (8.6)	1,562 (7.6)
**BMI (kg/m** ^2^ **)**
< 18.5	37,852 (2.6)	36,609 (2.7)	1,243 (1.2)	984 (1.2)	259 (1.3)
18.5–23.9	563,638 (39.4)	532,211 (40.0)	31,427 (31.4)	25,478 (32.0)	5,949 (29.0)
24.0–27.9	506,269 (35.4)	463,599 (34.8)	42,670 (42.6)	33,868 (42.5)	8,802 (42.9)
≥28.0	198,202 (13.8)	179,957 (13.5)	18,245 (18.2)	14,130 (17.7)	4,115 (20.1)
**Hypertension**
No	985,368 (68.8)	944,291 (70.9)	41,077 (41.0)	34,071 (42.7)	7,006 (34.1)
Yes	375,836 (26.3)	320,887 (24.1)	54,949 (54.8)	42,331 (53.1)	12,618 (61.5)
**Diabetes**
No	1,278,540 (89.3)	1,196,503 (89.9)	82,037 (81.8)	65,731 (82.4)	16,306 (79.5)
Yes	100,698 (7.0)	85,448 (6.4)	15,250 (15.2)	11,697 (14.7)	3,553 (17.3)
**Dyslipidemia**
No	857,214 (59.9)	802,817 (60.3)	54,397 (54.3)	43,071 (54.0)	11,326 (55.2)
Yes	526,230 (36.8)	483,075 (36.3)	43,155 (43.0)	34,557 (43.3)	8,598 (41.9)

BMI, body mass index; MRI, magnetic resonance imaging; SD, standard deviation.

^a^Data were presented as N (%) or mean ± SD, and all *P* < 0.001. There were 125,566 (8.8%), 70,323 (4.9%), 52,289 (3.7%), and 48,083 (3.4%) missing values for body mass index, hypertension, diabetes, and dyslipidemia, respectively.

Among participants with MRI-defined BI, the prevalence of hypertension, diabetes, and dyslipidemia were 54.8, 15.2, and 43.0%, respectively. The prevalence of hypertension, diabetes, and dyslipidemia among participants with MRI-defined BI was significantly greater in men than in women (*P* < 0.001; Table 1).

### Prevalence of MRI-defined brain infarcts

Of the 1,431,527 study participants, 100,245 (7.00%; 95% CI: 6.96–7.05%) were identified as having an MRI-defined BI. The mean age of participants with MRI-defined BI was 58.7 (SD 10.4) years and was similar among men and women: 58.7 (SD 10.6) years in men and 58.7 (SD 10.2) years in women. The crude prevalence of lacunar infarcts was 5.57% (*n* = 79,724, 95% CI: 5.53–5.61%) and non-lacunar infarcts was 1.43% (*n* = 20,521, 95% CI: 1.41–1.45%). The age- and sex-standardized prevalence of MRI-defined BI, lacunar infarcts, and non-lacunar infarcts were 5.79% (95% CI: 5.75–5.83%), 4.56% (95% CI: 4.52–4.60%), and 1.23% (95% CI: 1.21–1.25%), respectively (Table 2).

**Table 2:** The age- and sex-standardized prevalence of brain infarcts in the Chinese health examination population in 2018.

**Characteristics**	**Participants with MRI-defined brain infarcts**			**Participants with lacunar infarcts**			**Participants with non-lacunar infarcts**		
	**Total** **(*N* = 100,245)**	**Men** **(*N* = 56,196)**	**Women** **(*N* = 44,049)**	**Total** **(*N* = 79,724)**	**Men** **(*N* = 44,260)**	**Women** **(*N* = 35,464)**	**Total** **(*N* = 20,521)**	**Men** **(*N* = 11,936)**	**Women** **(*N* = 8,585)**
Total	5.79 (5.75–5.83)	6.30 (6.24–6.37)	5.28 (5.21–5.34)	4.56 (4.52–4.60)	4.93 (4.88–4.99)	4.19 (4.13–4.24)	1.23 (1.21–1.25)	1.37 (1.34–1.40)	1.09 (1.06–1.12)
**Age group**
18–29	0.46 (0.43–0.50)	0.51 (0.45–0.56)	0.42 (0.37–0.47)	0.42 (0.39–0.46)	0.46 (0.41–0.51)	0.38 (0.33–0.43)	0.05 (0.03–0.06)	0.05 (0.03–0.07)	0.04 (0.03–0.06)
30–39	0.98 (0.95–1.02)	1.06 (1.01–1.11)	0.90 (0.86–0.95)	0.85 (0.82–0.89)	0.91 (0.87–0.96)	0.79 (0.75–0.84)	0.13 (0.12–0.14)	0.14 (0.13–0.16)	0.11 (0.09–0.13)
40–49	3.40 (3.35–3.46)	3.91 (3.82–4.00)	2.88 (2.81–2.96)	2.87 (2.82–2.93)	3.24 (3.16–3.32)	2.50 (2.43–2.57)	0.53 (0.51–0.55)	0.67 (0.64–0.71)	0.38 (0.36–0.41)
50–59	9.39 (9.29–9.49)	10.62 (10.47–10.77)	8.13 (8.00–8.25)	7.62 (7.53–7.71)	8.54 (8.41–8.67)	6.67 (6.55–6.79)	1.77 (1.73–1.81)	2.08 (2.01–2.14)	1.46 (1.40–1.51)
60–69	19.52 (19.31–19.73)	21.12 (20.82–21.43)	17.87 (17.59–18.15)	15.03 (14.85–15.21)	16.07 (15.81–16.34)	13.96 (13.71–14.21)	4.49 (4.39–4.59)	5.05 (4.90–5.20)	3.91 (3.78–4.05)
70–79	30.30 (29.74–30.87)	31.47 (30.70–32.26)	29.09 (28.29–29.91)	22.68 (22.19–23.17)	23.41 (22.75–24.09)	21.92 (21.22–22.63)	7.62 (7.35–7.91)	8.06 (7.67–8.46)	7.17 (6.78–7.59)
≥80	37.33 (35.87–38.84)	39.05 (37.22–40.95)	35.56 (33.29–37.95)	27.71 (26.45–29.01)	29.46 (27.87–31.11)	25.91 (23.97–27.96)	9.62 (8.89–10.41)	9.60 (8.70–10.56)	9.65 (8.49–10.93)
**Geographical region**
Northeast	8.02 (7.87–8.18)	8.80 (8.58–9.03)	7.23 (7.02–7.45)	7.23 (7.08–7.38)	7.90 (7.69–8.11)	6.55 (6.35–6.76)	0.79 (0.74–0.84)	0.91 (0.84–0.98)	0.68 (0.61–0.75)
North China	6.57 (6.42–6.73)	7.47 (7.24–7.70)	5.68 (5.46–5.90)	3.94 (3.82–4.06)	4.58 (4.41–4.77)	3.30 (3.14–3.47)	2.63 (2.53–2.73)	2.88 (2.75–3.02)	2.38 (2.24–2.53)
Northwest	8.34 (8.17–8.51)	8.59 (8.37–8.82)	8.09 (7.84–8.35)	7.33 (7.18–7.50)	7.43 (7.22–7.64)	7.25 (7.01–7.49)	1.00 (0.94–1.07)	1.16 (1.08–1.25)	0.84 (0.76–0.93)
East China	4.63 (4.56–4.70)	4.89 (4.79–4.99)	4.36 (4.26–4.47)	3.58 (3.52–3.65)	3.77 (3.69–3.86)	3.39 (3.30–3.48)	1.05 (1.01–1.08)	1.12 (1.07–1.17)	0.97 (0.92–1.03)
Central China	6.47 (6.36–6.58)	7.24 (7.10–7.40)	5.69 (5.54–5.85)	4.72 (4.63–4.82)	5.26 (5.13–5.39)	4.19 (4.06–4.33)	1.74 (1.69–1.80)	1.99 (1.91–2.07)	1.50 (1.42–1.58)
South China	4.49 (4.35–4.63)	5.17 (4.97–5.37)	3.83 (3.64–4.03)	3.88 (3.75–4.01)	4.49 (4.31–4.68)	3.28 (3.11–3.46)	0.61 (0.56–0.67)	0.68 (0.60–0.75)	0.55 (0.48–0.64)
Southwest	4.02 (3.92–4.13)	4.35 (4.22–4.49)	3.69 (3.54–3.84)	3.28 (3.19–3.38)	3.51 (3.39–3.64)	3.05 (2.91–3.19)	0.74 (0.70–0.79)	0.84 (0.78–0.91)	0.64 (0.58–0.70)
**Body mass index (kg/m** ^2^ **)**
< 18.5	4.71 (4.43–5.00)	4.88 (4.48–5.29)	4.55 (4.17–4.96)	3.69 (3.44–3.95)	3.66 (3.32–4.02)	3.72 (3.37–4.09)	1.02 (0.89–1.16)	1.22 (1.02–1.43)	0.83 (0.67–1.02)
18.5–23.9	5.25 (5.18–5.33)	5.66 (5.56–5.76)	4.86 (4.75–4.96)	4.18 (4.12–4.25)	4.49 (4.40–4.58)	3.88 (3.80–3.97)	1.07 (1.04–1.11)	1.17 (1.13–1.22)	0.97 (0.92–1.02)
24.0–27.9	6.18 (6.10–6.26)	6.60 (6.50–6.70)	5.77 (5.65–5.89)	4.88 (4.81–4.95)	5.17 (5.08–5.26)	4.59 (4.48–4.70)	1.30 (1.27–1.34)	1.43 (1.38–1.48)	1.18 (1.12–1.23)
≥28.0	6.93 (6.80–7.07)	7.48 (7.30–7.67)	6.38 (6.18–6.58)	5.34 (5.22–5.46)	5.77 (5.61–5.93)	4.90 (4.72–5.08)	1.60 (1.53–1.66)	1.71 (1.63–1.80)	1.48 (1.39–1.58)
**Hypertension**
No	4.63 (4.56–4.71)	4.98 (4.89–5.08)	4.30 (4.19–4.40)	3.74 (3.68–3.80)	3.98 (3.90–4.06)	3.51 (3.41–3.60)	0.89 (0.86–0.93)	1.00 (0.96–1.05)	0.79 (0.74–0.84)
Yes	7.69 (7.57–7.80)	8.16 (8.04–8.27)	7.22 (7.02–7.43)	5.96 (5.86–6.06)	6.30 (6.20–6.41)	5.61 (5.44–5.80)	1.73 (1.68–1.79)	1.86 (1.80–1.91)	1.61 (1.51–1.71)
**Diabetes**
No	5.64 (5.59–5.69)	6.09 (6.03–6.16)	5.18 (5.11–5.25)	4.46 (4.41–4.50)	4.79 (4.73–4.85)	4.12 (4.06–4.19)	1.18 (1.16–1.21)	1.30 (1.27–1.33)	1.06 (1.03–1.10)
Yes	7.92 (7.53–8.32)	8.07 (7.75–8.41)	7.78 (7.08–8.56)	6.25 (5.87–6.64)	6.21 (5.92–6.52)	6.30 (5.61–7.07)	1.67 (1.58–1.78)	1.86 (1.73–2.02)	1.48 (1.37–1.66)
**Dyslipidemia**
No	5.56 (5.51–5.62)	6.07 (6.00–6.15)	5.06 (4.98–5.14)	4.36 (4.31–4.41)	4.73 (4.66–4.80)	3.99 (3.92–4.06)	1.21 (1.18–1.23)	1.35 (1.31–1.38)	1.07 (1.03–1.11)
Yes	6.20 (6.12–6.27)	6.64 (6.53–6.75)	5.76 (5.65–5.87)	4.92 (4.85–4.99)	5.24 (5.15–5.34)	4.60 (4.50–4.70)	1.28 (1.24–1.31)	1.39 (1.35–1.44)	1.16 (1.11–1.21)

Data are represented as percentage (95% confidence interval). The age- and sex-standardized prevalence of brain infarcts was calculated based on Chinese Census 2010.

The sex-standardized prevalence of MRI-defined BI was positively associated with age, ranging from 0.46% in 18–29 years and 0.98% in 30–39 years to 30.30% in 70–79 years and 37.33% for those ≥80 years. A particularly marked increase was noted among those 60 years or older (Table 2). The increasing trend with age was significant for both lacunar infarcts and non-lacunar infarcts. Men (6.30%; 95% CI: 6.24–6.37%) had a significantly greater age-standardized prevalence of MRI-defined BI than women (5.28%; 95% CI: 5.21–5.34%; *P* < 0.001), and the age-specific prevalence of MRI-defined BI was significantly higher among men than women across all age groups (Table 2).

**Figure 1:**
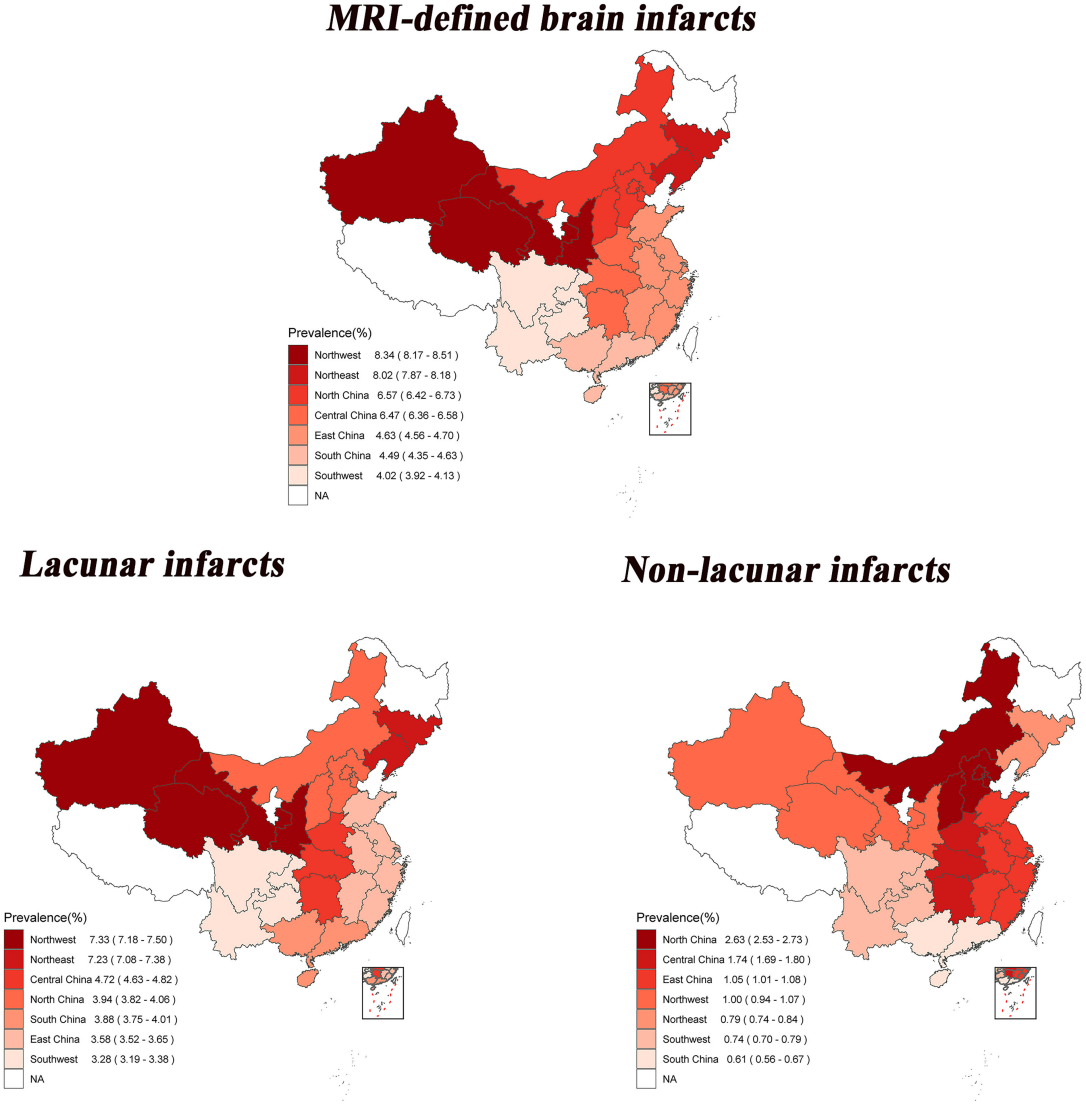
Age-and sex-standardized prevalence of brain infarcts among 28 provinces in China by geographical regions. The statistical data mentioned here do not include Heilongjiang, Ningxia, Tibet, Hong Kong, Macao and Taiwan.

The age- and sex-standardized prevalence of MRI-defined BI, lacunar infarcts and non-lacunar infarcts in seven major geographic regions are shown in Figure 1, depicting geographic variations in BI. In the seven geographic regions, the highest standardized prevalence of MRI-defined BI was observed in the Northwest (8.34%, 95% CI: 8.17–8.51%); followed by the Northeast (8.02%, 95% CI: 7.87–8.18%), and the lowest prevalence was observed in the Southwest (4.02%, 95% CI: 3.92–4.13%). The highest standardized prevalence of lacunar infarcts was also found in the Northwest (7.33%, 95% CI: 7.18–7.50%); followed by the Northeast (7.23%, 95% CI: 7.08–7.38%), and the lowest prevalence was observed in the Southwest (3.28%, 95% CI: 3.19–3.38%). The highest standardized prevalence of non-lacunar infarcts was observed in the North (2.63%, 95% CI: 2.53–2.73%); followed by the Central region (1.74%, 95% CI: 1.69–1.80%); and the lowest prevalence was observed in the South (0.61%, 95% CI: 0.56–0.67%).

### Multivariable analysis results

In the multivariable-adjusted analysis, men (OR, 1.17; 95% CI, 1.15–1.19), older age (OR per 10 year increment 2.33; 95% CI, 2.31–2.35), overweight (OR, 1.12; 95% CI, 1.10–1.14) or obesity (OR, 1.18; 95% CI, 1.16–1.21), hypertension (OR, 1.80; 95% CI, 1.77–1.83), diabetes (OR, 1.24; 95% CI, 1.21–1.26), and dyslipidemia (OR, 1.07; 95% CI, 1.05–1.08) were all significantly associated with higher risk of MRI-defined BI (*P* < 0.05; Table 3). The associations were also significant for both lacunar infarcts and non-lacunar infarcts, respectively.

**Table 3:** Multivariable adjusted odds ratios of MRI-defined brain infarcts in the Chinese health examination population in 2018.

**Characteristics**	**MRI-defined brain infarcts**		**Lacunar infarcts**		**Non-lacunar infarcts**	
	**Model 1**	**Model 2**	**Model 1**	**Model 2**	**Model 1**	**Model 2**
	**OR (95% CI)**	**OR (95% CI)**	**OR (95% CI)**	**OR (95% CI)**	**OR (95% CI)**	**OR (95% CI)**
Age (10-year increments)	2.52 (2.50–2.53)	2.33 (2.31–2.35)	2.33 (2.32–2.35)	2.18 (2.16–2.20)	2.48 (2.45–2.51)	2.30 (2.27–2.34)
**Sex**
Women	1.00	1.00	1.00	1.00	1.00	1.00
Men	1.26 (1.24–1.28)	1.17 (1.15–1.19)	1.22 (1.20–1.23)	1.13 (1.11–1.15)	1.32 (1.28–1.36)	1.25 (1.21–1.29)
**Geographical region**
Southwest	1.00	1.00	1.00	1.00	1.00	1.00
Northeast	2.49 (2.42–2.56)	2.38 (2.31–2.46)	2.75 (2.67–2.84)	2.63 (2.54–2.72)	1.09 (1.01–1.17)	1.06 (0.97–1.15)
North China	1.76 (1.70–1.82)	1.87 (1.80–1.94)	1.22 (1.17–1.26)	1.11 (1.06–1.16)	3.77 (3.55–4.00)	4.85 (4.53–5.20)
Northwest	2.67 (2.59–2.75)	2.81 (2.71–2.91)	2.82 (2.73–2.91)	2.99 (2.88–3.10)	1.47 (1.37–1.58)	1.37 (1.26–1.49)
East China	1.21 (1.18–1.24)	1.22 (1.18–1.26)	1.16 (1.12–1.19)	1.16 (1.12–1.19)	1.39 (1.31–1.47)	1.46 (1.37–1.56)
Central China	1.74 (1.69–1.79)	1.93 (1.87–1.99)	1.51 (1.46–1.56)	1.66 (1.60–1.72)	2.39 (2.25–2.53)	2.70 (2.52–2.88)
South China	1.17 (1.13–1.21)	1.30 (1.25–1.35)	1.25 (1.20–1.29)	1.35 (1.29–1.40)	0.84 (0.77–0.91)	1.01 (0.92–1.11)
**Body mass index (kg/m** ^2^ **)**
< 24	1.00	1.00	1.00	1.00	1.00	1.00
24.0–27.9	1.28 (1.26–1.30)	1.12 (1.10–1.14)	1.25 (1.23–1.27)	1.11 (1.09–1.13)	1.36 (1.32–1.41)	1.15 (1.11–1.20)
≥28.0	1.52 (1.49–1.55)	1.18 (1.16–1.21)	1.43 (1.40–1.46)	1.15 (1.12–1.18)	1.75 (1.68–1.83)	1.29 (1.23–1.35)
**Hypertension**
No	1.00	1.00	1.00	1.00	1.00	1.00
Yes	1.91 (1.88–1.94)	1.80 (1.77–1.83)	1.77 (1.74–1.79)	1.67 (1.64–1.70)	2.24 (2.17–2.31)	2.07 (2.00–2.14)
**Diabetes**
No	1.00	1.00	1.00	1.00	1.00	1.00
Yes	1.39 (1.36–1.42)	1.24 (1.21–1.26)	1.32 (1.29–1.35)	1.20 (1.17–1.23)	1.48 (1.43–1.55)	1.27 (1.21–1.32)
**Dyslipidemia**
No	1.00	1.00	1.00	1.00	1.00	1.00
Yes	1.21 (1.19–1.23)	1.07 (1.05–1.08)	1.22 (1.20–1.24)	1.07 (1.05–1.09)	1.14 (1.11–1.18)	1.07 (1.04–1.11)

Model 1 adjusted for age (10 year increments) and sex.

Model 2 adjusted for all co-variables listed in the table. ORs of 1.00 indicate reference values.

## Discussion

Among this population of 1.4 million participants for health examinations across China, we found that the age- and sex-standardized frequency of MRI-defined BI was 5.79% (95% CI, 5.75–5.83%) in 2018, and the majority of these infarcts was lacunar infarcts. We observed that the sex-standardized frequency of MRI-defined BI is strongly associated with increasing age. Sex differences and geographical variations in the frequency of MRI-defined BI were also observed in our study with greater frequency in men and in northern regions. Overweight/obesity, hypertension, diabetes, and dyslipidemia were significantly positively associated with the risk of MRI-defined BI. To the best of our knowledge, this study is the largest investigation of the distribution and risk factors of BI detected by MRI. Our findings provide solid evidence of a substantial burden of MRI-defined BI in the health examination population.

Previous studies have reported the prevalence of BI with a wide range globally (7, 10, 17). A systematic review of 27 studies showed that the prevalence of BI in most studies ranged from 10 to 20% (10). The sample size of these studies ranged from 219 to 3,397, with the mean age ranging from 49 to 79 years (10). Most of the studies (20 of 27 studies) were conducted among participants over 60 years of age. In the Rotterdam Scan Study of 1,077 community residents (mean age of 72 years), Vermeer et al. (6) observed that 217 (20%) participants had SBI. In a survey of 994 Korean adults (mean 49.0 years of age) who underwent routine health examinations, SBI lesions were observed in 58 (5.84%) participants (18). The age- and sex-standardized prevalence of MRI-defined BI in our study (5.79%) was lower than most previous studies, largely due to relatively healthy health examination participants in our study (mean age of 46.4 years).

Although the reported prevalence of BI varies widely in previous studies, the prevalence is significantly higher among older individuals, which is consistent with findings from our study (7, 10). The SBI prevalence in the Rotterdam Scan Study increased from 8% in the 60–64 age group to 35% among those ≥80 years (6). In the study based on health examination data from Korean adults, Lee et al. (18) observed no SBI among those aged 20–39 years, however, SBI prevalence increased to 1.7% among those aged 40–49 years and to 43.8% in those aged 70–79 years. MRI-defined BI is considered a common radiological finding among the older population. However, in our study with a large sample size and a wide range in age, a proportion of MRI-defined BI was detected even among the younger population (prevalence of MRI-defined BI was 0.46% in 18–29 years, 0.98% in 30–39 years and 3.40% in 40–49 years). Previous epidemiological studies have shown a rising trend of overt stroke among younger age groups in recent years, which may drive morbidity and mortality among young and middle-age groups, posing a substantial burden to health-care systems and the economy due to the long-lasting consequences (19). As an early predictor of overt stroke, covert MRI-defined BI should not be neglected, especially among young adults. More effective guidelines and policies are needed to prevent and manage clinically unrecognized BI (7).

Our study suggests the significant association of hypertension, diabetes, and other metabolic disorders with BI. Apart from age, hypertension has been widely accepted as a risk factor for BI (10). Previous studies have suggested that hypertension plays an important role in the pathogenesis of BI (10). A meta-analysis found a significant association between diabetes and the risk of MRI-defined BI (7), which is consistent with our findings. However, the association between being overweight or obese with a risk of BI has been controversial with inconsistent results reported in previous studies (10). These inconsistencies might be due to the limitations of BMI for defining overweight or obesity, which does not distinguish between fat and lean mass (20, 21). Inconsistent findings have also been reported for the association between dyslipidemia (total cholesterol, high-density lipoproteins, low-density lipoproteins, and triglycerides) and BI (7, 10). Chauhan et al. (7) have concluded that a higher risk of MRI-defined BI was significantly associated with higher triglyceride levels but no association was observed with cholesterol levels. Triglyceride levels have been associated with inflammatory markers, blood-brain barrier dysfunction, β-amyloid synthesis, and the promotion of β-amyloid delivery to the brain, which could contribute to the pathogenesis of the cerebrovascular disease (22–24). In two large French population-based studies, no association between cholesterol levels and MRI markers of cerebral small vessel disease, white matter hyperintensity volume, and lacunes was found (24). In contrast to these previous studies limited by small sample sizes, our study with a substantially large sample size had the statistical power to detect associations between MRI-defined BI and metabolic risk factors including among different sub-groups.

Our study showed geographical variation in the epidemiology of MRI-defined BI in China, with the highest prevalence of BI observed in the northern and central regions, which is consistent with a nationally representative study of stroke burden (25). Geographical variations in BI burden may be related to differences in risk factors for BI across these regions. As our study observed that hypertension, diabetes, and obesity were associated with a higher risk of MRI-defined BI, we also observed that the highest prevalence of hypertension, diabetes, and obesity were reported in the northern compared to other regions (26–28). Geographical variations in BI burden might be partly attributable to location-associated lifestyle and genetic background (7, 29). Finally, lower socioeconomic status and poor access to health care services have been associated with a higher risk of cerebrovascular disease (30), and differences in socioeconomic status between these regions might have contributed to geographical variations in BI burden. Geographical variations in BI imply that specific geographical regions should prioritize the allocation of healthcare resources. It is crucial to track spatial trends in the BI burden to reduce geographical disparities in BI. The finding that geographical distribution differed between lacunar and non-lacunar brain infarcts could be explained by differences in distinct etiology and risk factors between lacunar and non-lacunar brain infarcts (23, 31, 32), which warrants further investigation.

This study has several strengths. First, our study was the largest survey to date to assess the burden of MRI-defined BI and provided sufficient power to examine the prevalence of MRI-defined BI in a wide variety of subgroups. Second, the wide range in age of participants in our study allowed us to evaluate the burden of MRI-defined BI in the younger adults, which was not feasible in previous studies that primarily focused on older participants. Finally, to the best of our knowledge, our study was the first investigation to evaluate the geographical variation of MRI-defined BI nationwide, which provides vital information for allocating healthcare resources from a multilevel geographical perspective to reduce the burden of MRI-defined BI.

Our study had several potential limitations. First, we did not systematically collect information about the education level, income level, smoking status, alcohol consumption, diet, or physical activity of health examination participants. This limited our ability to explore these potential risk factors in association with MRI-defined BI. Second, data on the history of clinically defined stroke or stroke symptoms were not collected in our study, thus we did not distinguish between SBI and clinical stroke in the study. Previous studies have shown that the vast majority of MRI-defined BI were SBI, especially in the preventive health examination population (7, 10, 17). Third, because the MRI scan is relatively expensive, the socioeconomic status of our population may have left out the group whose socioeconomic status is relatively low. Furthermore, the participants who received health examinations in our study cannot represent the overall Chinese population due to available data from real-world health screening practices.

In conclusion, the study indicates that MRI-defined BI is highly prevalent among the health examination population in China and that MRI-defined BI is also prevalent among younger adults. The prevalence is higher among men than women and in the northern and central regions of the country. Overweight/obesity, hypertension, diabetes, and dyslipidemia are preventable risk factors for MRI-defined BI. Public health strategies that consider sex and geographic disparities are needed to develop BI prevention strategies in China.

## Data availability statement

The data that support the findings of this study are available from the corresponding author upon reasonable request.

## Ethics statement

The studies involving human participants were reviewed and approved by Peking University Institutional Review Board (ID: IRB00001052-19077). Written informed consent from the participants' legal guardian/next of kin was not required to participate in this study in accordance with the national legislation and the institutional requirements.

## Author contributions

LL, YN, and BW contributed significantly to conceive and designed the study and revised the manuscript. JW and YG did the statistical analysis. JW drafted the manuscript. All authors contributed to interpreted data, critical revisions, read, and agreed to the published version of the manuscript.

## Funding

This work was supported by the National Natural Science Foundation of China (Grants 82192901, 82192900, and 91846303) and the National Key R&D Program (Grant 2020YFC2003400).

## Conflict of interest

The authors declare that the research was conducted in the absence of any commercial or financial relationships that could be construed as a potential conflict of interest.

## Publisher's note

All claims expressed in this article are solely those of the authors and do not necessarily represent those of their affiliated organizations, or those of the publisher, the editors and the reviewers. Any product that may be evaluated in this article, or claim that may be made by its manufacturer, is not guaranteed or endorsed by the publisher.

## Author disclaimer

The content is solely the responsibility of the authors and does not necessarily represent the official views of the sponsoring institutions.

## Abbreviations

ANOVA: analysis of variance
BMI: body mass index
BI: brain infarcts
CI: confidence interval
MRI: magnetic resonance imaging
OR: odds ratio
SBI: silent brain infarcts
SD: standard deviation.
